# Promoterless Gene Targeting Approach Combined to CRISPR/Cas9 Efficiently Corrects Hemophilia B Phenotype in Neonatal Mice

**DOI:** 10.3389/fgeed.2022.785698

**Published:** 2022-03-11

**Authors:** Michela Lisjak, Alessia De Caneva, Thibaut Marais, Elena Barbon, Maria Grazia Biferi, Fabiola Porro, Adi Barzel, Lorena Zentilin, Mark A. Kay, Federico Mingozzi, Andrés F. Muro

**Affiliations:** ^1^ International Centre for Genetic Engineering and Biotechnology, Trieste, Italy; ^2^ Inserm UMRS974, Centre of Research in Myology (CRM), Institut de Myologie, Sorbonne Université, Paris, France; ^3^ Genethon, Evry, France; ^4^ IRCCS San Raffaele Hospital, Milan, Italy; ^5^ Department of Biochemistry and Molecular Biology, The George S. Wise Faculty of Life Sciences, Tel Aviv University, Tel Aviv, Israel; ^6^ Departments of Pediatrics and Genetics, Stanford University, Stanford, CA, United States; ^7^ University Pierre and Marie Curie - Paris 6, INSERM U974, Paris, France; ^8^ Spark Therapeutics, Philadelphia, PA, United States

**Keywords:** GeneRide, albumin gene targeting, mouse model, coagulation factor IX, tail clip test, CRISPR/SaCas9

## Abstract

Many inborn errors of metabolism require life-long treatments and, in severe conditions involving the liver, organ transplantation remains the only curative treatment. Non-integrative AAV-mediated gene therapy has shown efficacy in adult patients. However, treatment in pediatric or juvenile settings, or in conditions associated with hepatocyte proliferation, may result in rapid loss of episomal viral DNA and thus therapeutic efficacy. Re-administration of the therapeutic vector later in time may not be possible due to the presence of anti-AAV neutralizing antibodies. We have previously shown the permanent rescue of the neonatal lethality of a Crigler-Najjar mouse model by applying an integrative gene-therapy based approach. Here, we targeted the human coagulation factor IX (hFIX) cDNA into a hemophilia B mouse model. Two AAV8 vectors were used: a promoterless vector with two arms of homology for the albumin locus, and a vector carrying the CRISPR/SaCas9 and the sgRNA. Treatment of neonatal P2 wild-type mice resulted in supraphysiological levels of hFIX being stable 10 months after dosing. A single injection of the AAV vectors into neonatal FIX KO mice also resulted in the stable expression of above-normal levels of hFIX, reaching up to 150% of the human levels. Mice subjected to tail clip analysis showed a clotting capacity comparable to wild-type animals, thus demonstrating the rescue of the disease phenotype. Immunohistological analysis revealed clusters of hFIX-positive hepatocytes. When we tested the approach in adult FIX KO mice, we detected hFIX in plasma by ELISA and in the liver by western blot. However, the hFIX levels were not sufficient to significantly ameliorate the bleeding phenotype upon tail clip assay. Experiments conducted using a AAV donor vectors containing the eGFP or the hFIX cDNAs showed a higher recombination rate in P2 mice compared to adult animals. With this study, we demonstrate an alternative gene targeting strategy exploiting the use of the CRISPR/SaCas9 platform that can be potentially applied in the treatment of pediatric patients suffering from hemophilia, also supporting its application to other liver monogenic diseases. For the treatment of adult patients, further studies for the improvement of targeting efficiency are still required.

## Introduction

Hemophilia B is a serious X-linked recessive bleeding disorder caused by mutations in the coagulation factor IX (FIX) gene (Mannucci and Tuddenham, 2001). The current therapy is based on the regular infusion of recombinant FIX. However, this treatment still suffers from several limitations, such as the need for life-long repeated intravenous infusions of the recombinant factor, reducing the life quality of the patients. Moreover, a significant proportion of patients develop anti-drug antibodies induced by the recombinant FIX protein, which considerably reduce therapeutic efficacy (DiMichele, 2007; Weyand and Pipe, 2019).

Gene therapy approaches represent a promising strategy for hemophilia B since relatively low levels of factor IX are required to increase the coagulation efficiency, reducing bleeding episodes. Indeed, recurrent spontaneous hemarthrosis episodes frequently observed in severe and moderate forms of the diseases (<1%, and 1% to <5% of residual factor IX activity, respectively), are rare when coagulation factor IX activity is higher than 5%, as observed in mild cases (5 to <30% of the normal FIX value) (Mannucci and Tuddenham, 2001).

Adeno-associated viral (AAV) vectors have shown high potentiality for *in vivo* liver gene transfer proving long-term efficacy in pre-clinical studies with animal models and adult patients (Wang et al., 1997; Wang et al., 2000; Mount et al., 2002; Nathwani et al., 2011; Nathwani et al., 2014; George et al., 2017; Rangarajan et al., 2017; Pasi et al., 2020). This strategy is based on the delivery of an expression cassette that remains in an episomal form, in which the therapeutic cDNA is under the transcriptional control of a liver-specific promoter. However, this approach has important limitations for the treatment of neonate and pediatric patients, and certain disease states, characterized by a high rate of hepatocyte duplication (Colella et al., 2018) leading to the loss of vector DNA and reduction of the therapeutic efficacy (Cunningham et al., 2008; Wang et al., 2012; Bortolussi et al., 2014). On the other hand, vector re-administration may be limited by the long-term presence of high titers of neutralizing antibodies against the AAV capsid, which are generated during the first administration (Nathwani et al., 2014; George et al., 2020). Thus, new therapeutic approaches are needed for the treatment of newborn and pediatric patients.

During the last years, the use of genome-editing approaches has exponentially increased (Carroll, 2014; Cox et al., 2015; Tong et al., 2019; Trevisan et al., 2020). The main advantage is the permanent modification of the target-cell genome assuring long-term correction. This approach is efficient also in tissues with active proliferation such as the neonatal/pediatric liver, since the targeted allele is stably transmitted to daughter cells. Thus, as an alternative approach to gene replacement, we performed a genome targeting strategy also called “GeneRide”, based on the insertion of a promoterless therapeutic cDNA into the 3′ coding region of the Albumin (Alb) gene, without the use of engineered nucleases (Barzel et al., 2015). The targeted transgene remains under the transcriptional control of the strong albumin promoter. Transcription of the targeted allele results in a single chimeric mRNA containing the complete albumin ORF, a self-cleaving peptide (P2A), which is translated into two separate proteins (Barzel et al., 2015).

We have demonstrated that AAV-mediated gene targeting of a promoterless UGT1A1 cDNA into the albumin locus without the use of nucleases was able to rescue neonatal lethality in a mouse model of the Crigler-Najjar syndrome lowering bilirubin to life-compatible levels (Porro et al., 2017). To increase the recombination rate and therapeutic efficacy in neonates, we combined GeneRide with the site-specific engineered endonuclease CRISPR/SaCas9. This resulted in the complete rescue of neonatal mortality and long-term complete correction of the disease phenotype by decreasing plasma bilirubin to wild-type levels (De Caneva et al., 2019).

In the present work, we demonstrate that the combination of GeneRide with SaCas9 in neonatal WT and hemophilia B mice resulted in the long-term production of supraphysiological levels of human FIX, leading to a normal coagulation process. Despite the potential of the treatment in neonates, it was apparently less effective when applied to adult hemophilic mice.

## Materials and Methods

### Animals

Mice were housed and handled according to institutional guidelines. FVB neonate mice were used for eGFP experiments and were held at ICGEB Bioexperimental facility in Trieste, Italy. Experimental procedures were approved by the International Centre for Genetic Engineering and Biotechnology (ICGEB) board and by the Italian Ministry of Health (authorization N. 996/2017-PR from the Italian Ministry of Health). All experiments involving animals were conducted in full respect of the ARRIVE principles. FIX KO mouse model (strain B6.129P2-*F9<*tm1Dws>/J) was used for Hemophilia B treatments. Mice were held and treated at the Sorbonne University—UMS028, (105, boulevard de l’Hôpital, Paris). Animals were maintained following the French and European guidelines for the use of animal models (2010/63/EU). The experimental protocol was approved by the Charles Darwin N.005 Ethical Committee on Animal Experiments (number 22204). Mice were kept in a temperature-controlled environment with a 12–12 h light-dark cycle. They received a standard chow diet and water ad libitum.

### rAAV Vectors Production

The recombinant AAV vectors used in this study are based on AAV type 2 backbone and were prepared by AAV Vector Unit at ICGEB Trieste (https://www.icgeb.org/avu-core-facility.html) in HEK293 cells by a cross-packing approach whereby the vector was packaged into AAV capsid 8, as described (Bortolussi et al., 2014).

### rAAV Treatment of Wild-Type, Neonatal and Adult FIX^−/−^ Mice

For AAV-eGFP-donor vector treatment, P60 wild-type mice were injected intravenously with rAAV-donor-EGFP (5.0E11 vg/mouse, *n* = 3) with or without the rAAV-SaCa9-sgRNA8 (1.0E11 vg/mouse, *n* = 3). Mice were sacrificed after 2 weeks (+SaCas9) or 1 month (−SaCas9).

FIX^−/−^ mice were treated with a donor vector encoding for hFIX protein and the SaCRISPR/Cas9 system. A neonatal mouse group was injected at post-natal day two and the adult group at 2 months of age. Neonatal mice were injected intravenously at P2 with rAAV8-SaCas9 (0.6E11 vg/mouse) and rAAV8-donor-hFIX (5.0E11 vg/mouse), 1:8 ratio (*n* = 12). Mice were sacrificed at 4 months of age. Mice injected at post-natal day 60 (2 months old) in a 1:5 ratio of rAAV8-SaCas9 (1.0E11 vg/mouse) and rAAV8-donor-hFIX (5.0E11 vg/mouse) and sacrificed 2 months after the treatment at 4 months of age (*n* = 8).

### Tail Clip Test

The tail clip test on FIX^−/−^ mice was slightly modified from Liu et al. (Liu, 2012). Briefly, animals were anesthetized with a mix of ketamine (Imalgene) 100 mg/kg and xylazine (Rompun) 20 mg/kg. During anesthesia, a 3 mm piece of the tail tip was cut with a scalpel and 2 cm of the tail extremity was placed in a 50 ml Falcon tube containing 50 ml of PBS, pre-warmed at 37°C. Bleeding was monitored for 20 min (min). After 20 min, the Falcon tubes were centrifuged for 5 min at 4.000 rpm, and the PBS was carefully removed. Cell pellet was then hemolyzed with 1 ml of Red Blood Cells lysis buffer (Qiagen). Lysates were then transferred in 2 ml Eppendorf tubes and centrifugated for 10 min at 10.000 rpm. The optical density of supernatants was then measured using a plate reader (Spark, Tecan Life Science) at 550 nm. The final blood loss per mouse was compared to a standard curve, obtained from wild-type mice subjected to the same procedure.

### Plasma hFIX and Anti-hFIX Antibody Determination

hFIX levels were analyzed by enzyme-linked immunosorbent assay (ELISA), using an antibody set FIX-EIA provided by Enzyme Research Laboratories (United States) following the manufacturer’s protocol. Briefly, a 96-well plate was coated with a capture antibody (anti-hFIX) for 2 h. After washing, samples were diluted 1/1,000 or 1/25 in sample diluent while reference plasma was serially diluted starting from 1/100 (100%) to 1/3,200 (3,13%) and left incubated for 90 min. After another washing step, a polyclonal goat anti-human F9 peroxidase-conjugated IgG secondary antibody (Enzyme Research GAFIX-APHRP). After the washing step, to develop the color, OPD substrate was used and the reaction was stopped by adding 2.5 M H_2_SO_4._ The plate was read at a wavelength of 490 nm on a multi-plate reader (Perkin Elmer Envision Plate Reader, Walthman, MA). The hFIX levels were calculated considering reference values of 5,000 ng/ml.

To evaluate the presence of anti-hFIX antibodies, we followed a previously described protocol (Meliani et al., 2017). We coated the plate with 1 µg of native hFIX protein (Invitrogen), mouse IgG (Millipore) to generate a standard curve (starting from 100 ng/μl) left incubating overnight. After the blocking step with (2% bovine serum albumin in PBS-0.05% Tween-20), we diluted the plasma sample in 1:10 dilution and detected it with an anti-mouse IgG-HRP antibody. The detection was performed as described above. For the positive control, we incubated the hFIX protein with an anti-hFIX antibody (Sigma Aldrich).

### Genomic DNA Extraction From Liver Tissue and Viral Genome Copies Quantification

The extraction kit used for this procedure is Wizard^®^ SV Genomic DNA Purification System by Promega following the manufacturer’s instructions. The viral genome copies were analyzed as described previously (De Caneva et al., 2019). Primer set for rAAV8 pAB vector: forward (5′- ACT​TCT​TGT​CTC​TGT​GCT​GC-3′) and reverse (5′-TGA​TTA​ACC​CGC​CAT​GCT​AC-3′). For rAAV8 Cas9 vector: forward (5′-AAG​GAT​CAC​CCA​GCC​TCT​GC-3′) and reverse (5′- CCT​GCT​GAA​GAC​ACT​CTT​GCC​A-3′).

### ddPCR

The on-target recombination rate was measured using ddPCR of liver genomic DNA as described previously (Tsuji et al., 2022). Briefly, 100 ng or 200 ng of gDNA were digested with SpeI for 1 h. 25 ng of digested gDNA was added to a 25 μl PCR reaction containing ddPCR Supermix for Probes (No dUTP) (Bio-Rad), 900 nM target-specific primers, and 250 nM amplicon-specific probes. Droplets were generated using 22 μl of PCR reactions and 70 μl of oil according to manufactures instructions. Reactions were cycled as follows: 95°C for 10 min, 50 cycles of 95°C for 30 s, 60°C for 30 s, and 72°C for 6 min, and one cycle of 95°C for 10 min and held at 4°C until droplet reading. Primers to amplify a 1.6 kb non-targeted region of endogenous mouse Albumin were (5′-CTG​CTG​TGC​ACC​AGT​TGA​TGT​T-3′) and (5′- TGC​TTT​CTG​GGT​GTA​GCG​AAC​T-3′), combined with a HEX-labeled probe (5′- TCT​GGT​GCT​GAG​GAC​ACG​TAG​CCC​AGT -3′). Primers to amplify on-target HR with a 1.4 kb amplicon were (5′-GGG​CAA​GGC​AAC​GTC​ATG​G-3′) and (5′- CCA​GGG​TTC​TCT​TCC​ACG​TC-3′), combined with a FAM-labeled probe (5′-GCCCAAGGCTAC AGCGGAGC-3′).

### Total RNA Extraction and mRNA Expression Analysis

RNA was extracted by homogenizing liver powder in NucleoZOL solution (Takarabio) following the supplier’s instructions. The cDNA was retro-transcribed using M-MLV reverse transcriptase (Invitrogen, Carlsbad, CA, United States). mRNA expression was evaluated as previously described (De Caneva et al., 2019) using a primer set specific for mAlb-hFIX fusion mRNA (forward 5′-AAG​GCA​CCA​GCT​TTC​TGA​CC-3′ and reverse 5′- TGA​GTC​CTG​AGT​CTT​CAT​GTC​TT-3′). For endogenous mFIX, the primers were specific for mouse FIX (forward 5′- TTC​CTA​TGA​ATG​CTG​GTG​CCA​AG-3′ and reverse 5′-CTG​TTG​GTT​CAC​AGG​ACT​TCT​GG-3′).

### RT-qPCR Primer Efficiency

Primer efficiency was compared by amplifying by PCR the chimeric hFIX-Alb in treated mice and endogenous mFIX in wild-type mice. The amplicons were quantified with nanodrop and a qPCR was performed using serial dilutions (1:10) of the amplicon starting from 0.2 ng of template. The Cq mean values and the template quantity of the chimeric mRNA in treated mice and the endogenous mRNA levels of wild-type mice were plotted in a graph.

### Total Protein Liver Extraction and Western Blot Analysis

Total protein extracts were obtained by homogenizing 10 mg of liver in 200 µl of lysis buffer (50 mM Hepes pH = 7.4, 150 mM NaCl, 1 mM EDTA, 0.5% NP40). Total protein was quantified using Bradford (Biorad). 40 µg of protein was used for analyzing eGFP and 5 µg or 50 µg for hFIX in neonatal or adult studies. Plasma was diluted at 1:50 for western blot analysis in plasma. Primary and secondary antibodies were used as described in Supplementary Table S1.

### Immunostaining of hFIX in Liver Tissue

Liver lobes from FIX KO were dissected and frozen in liquid nitrogen. Later they were cut at 8 µm slices in a cryostat. Sections were thawed at room temperature and rinsed in PBS ×1. Fixation and permeabilization step was performed in acetone for 10 min at −20°C. Slices were washed three times with PBS ×1 for 5 min. Slices were blocked for 90 min in a blocking solution containing 10% normal goat serum (NGS) Triton 0.1%, PBS ×1. The primary antibody (GAFIX-AP, Affinity Biologicals) was diluted 1/50 in blocking solution and left incubating overnight at 4°C. The next day samples were washed 3 times in PBS ×1 for 5 min. Slides were incubated with the secondary antibody AlexaFluor 647 IgG at 1/500 in Triton 0.1%, PBS ×1 for 1 h. After washing the slices 3 times with PBS ×1, slices were incubated with Hoechst 1:5000 in PBS ×1 for 10 min and washed 3 times in PBS ×1 for 5 min. After a rinse in water, slices were covered with a coverslip in mowiol.

For eGFP detection, liver lobes were fixed in 4%PFA in PBS ×1 for 24 h at 4°C and the PFA was then changed with 20% sucrose 0.02% sodium azide in PBS ×1 and kept at 4°C. Liver lobes were cut in a cryostat in 4 μm slices and washed three times with PBS ×1 and stained with Hoechst solution for 10 min.

Fluorescent images were taken with a Nikon fluorescence microscope. Images were modified using ImageJ software version 2.0.0-rc-69/1.52p.

### Statistics

Statistical analyses were performed using GraphPad Prism 8.2.1. Data in the graphs are expressed as means ± standard error of the mean (SEM) or standard deviation (SD), as indicated. The student’s *t*-test was used for analyzing two groups. For comparing more than two groups, one-way or two-way ANOVA was used followed by indicated post-hoc test. A *p* value < 0.05 was considered statistically significant.

## Results

**FIGURE 1:**
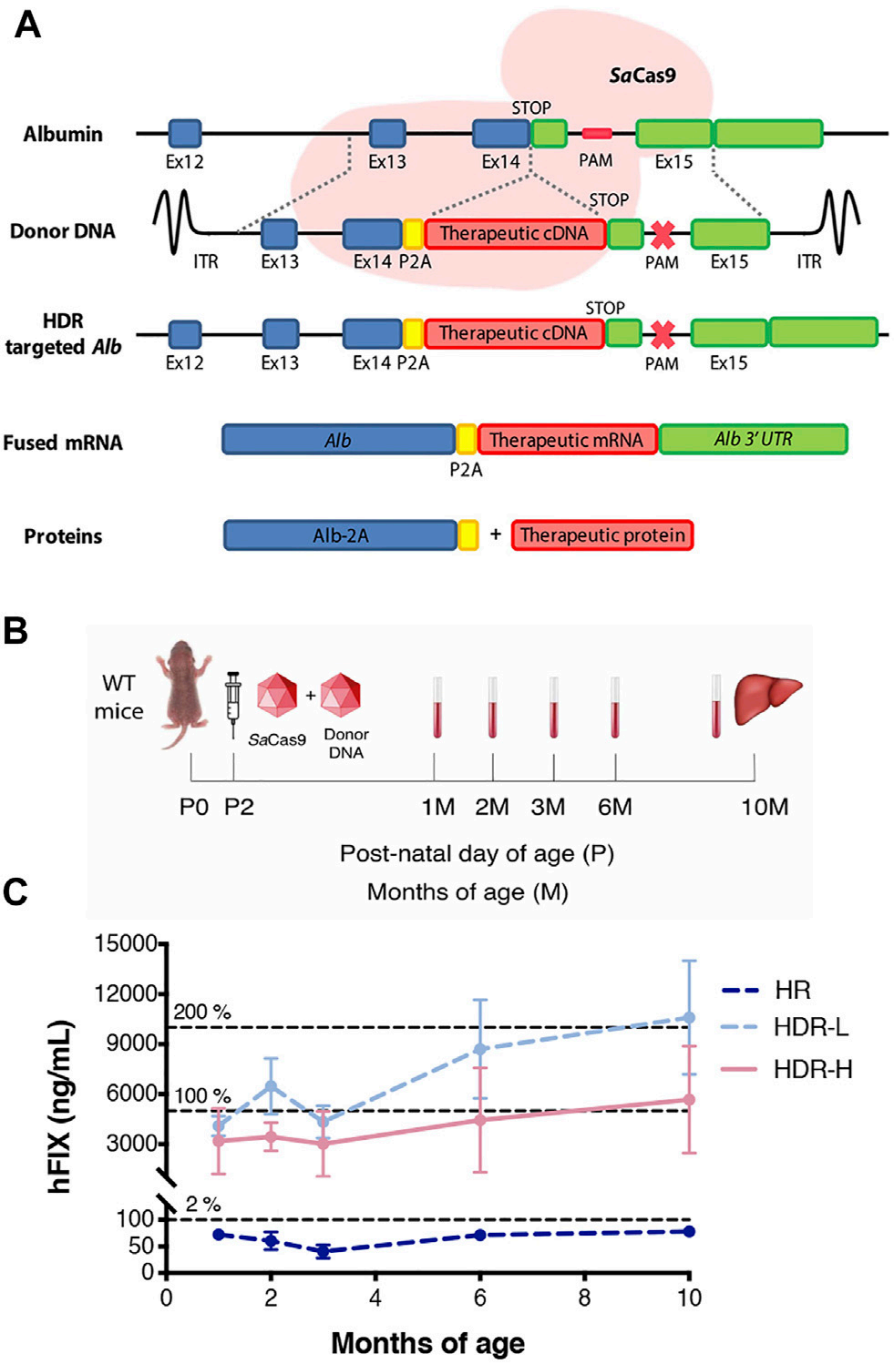
*In vivo* gene targeting of hFIX into the albumin locus in neonatal wild-type mice. **(A)** Targeting strategy for integration of donor hFIX cDNA vector, proceeded by the 2A-peptide and flanked with arms of homology for the albumin locus. Albumin and hFIX are transcribed into a single hybrid mRNA molecule and translated into two separate proteins. The CRISPR/*Sa*Cas9 performs a double-strand break in the intron located downstream of the albumin stop codon, enhancing the homologous directed repair rate; **(B)** Experimental scheme. Wild-type mice were i.v. injected at post-natal day 2 with only rAAV8-donor-hFIX (2.0E11 vg/mouse, HR, *n* = 5) or with rAAV8-donor-hFIX combined to different rAAV8-*Sa*Cas9-sgRNA8 doses (6.0E10 vg/mouse, HDR L, *n* = 5; or 2.0E11 vg/mouse, HDR H, *n* = 5). Blood was collected at different time points and mice were sacrificed at 10 months of age; **(C)** Plasma hFIX levels were evaluated at 1, 2, 3, 4, 6, and 10 months of age. The dotted lines at 5,000 ng/ml and 10,000 ng/ml represent 100 and 200%, respectively, of the hFIX levels present in the healthy human population. Values are represented with mean ± SD. Two-way ANOVA (Bonferroni test) Interaction ns (*p* = 0.1356), Treatment *** (*p* < 0.0001), Time ** (*p* = 0.0074).

Aiming at developing a highly efficient gene therapy for hemophilia B, we constructed a promoterless donor vector containing the human coagulation factor IX cDNA, carrying the V86A, E277A, and R338L-Padua hyperactive mutations (Simioni et al., 2009; Lin et al., 2010; Kao et al., 2013). This resulted in the integration of the hFIX cDNA after the albumin ORF, upstream to the stop codon. A chimeric mRNA is transcribed and, due to the presence of the teschovirus 2A peptide (P2A) between both ORFs (Barzel et al., 2015), two separate proteins, albumin and a hyperactive FIX, can be translated (Figure 1A). The donor construct was mutated at the sgRNA PAM site to avoid Cas9 cleavage of the donor DNA or the targeted allele.

To determine the efficacy of the strategy, we first treated WT FVB neonate male mice (at post-natal day 2, P2) with an intravenous injection of the promoterless hFIX-donor DNA (rAAV8-donor-hFIX, 2.0E11 vg/mouse, *n* = 5) alone, or in combination with the SaCas9-sgRNA8 encoding vector using two different donor/Cas9 vector ratios [rAAV8-SaCas9-sgRNA8, 6.0E10 or 2.0E11 vg/mouse, for the low (HDR L, *n* = 5) and high (HDR H, *n* = 5) SaCas9 dose, respectively]. Blood from rAAV8-treated and untreated mice was collected by retro-orbital bleeding at 1, 2, 3, 6, and 10 months, which was the last analyzed time-point (Figure 1B). One month after the treatment, the levels of hFIX in the animals dosed with donor vector and SaCas9 were already in the range of those present in the human population, while those of the group treated only with the donor vector (no SaCas9) were about 50-fold lower (Figure 1C). We observed no direct correlation between the hFIX plasma levels and the dose of the rAAV8-SaCas9-sgRNA8 vector. In fact, the levels of plasmatic hFIX in the HDR-L group of animals reached ∼10,000 ng/ml at 10 months of age, while they were ∼6,000 ng/ml in the HDR-H group, although these differences did not reach statistical significance.

**FIGURE 2:**
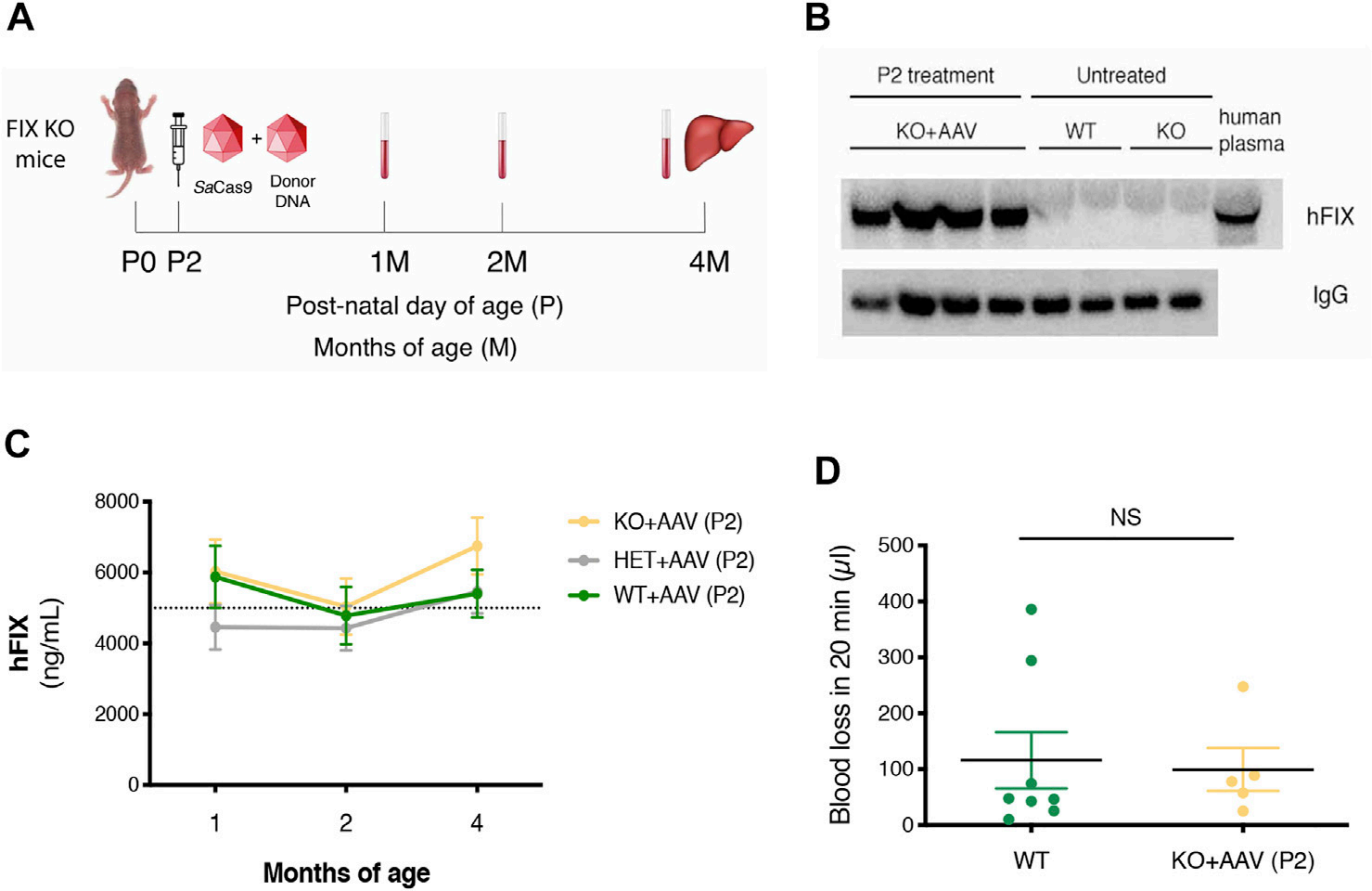
Targeting the hFIX cDNA into the albumin locus of neonatal FIX KO mice. **(A)** Experimental design. FIX KO neonatal mice were injected at postnatal day (P) 2 with 0.6E11 vg/mouse of AAV8-SaCas9 and 5.0E11 vg/mouse of AAV8-donor-hFIX. Bleeding was performed at 1, 2 months (M) and mice were sacrificed at 4 months. The liver was collected for molecular analysis; **(B)** hFIX plasma levels (ng/ml) at 1, 2, and 4 months of age in WT (*n* = 11), Het (*n* = 6) and FIX KO (*n* = 15) mice transduced with *Sa*Cas9 and donor-hFIX treated at post-natal (P) day 2. 5,000 ng/ml corresponds to the normal FIX plasma levels in healthy individuals in the human population; **(C)** Western blot analysis of plasma hFIX in treated FIX KO mouse plasma. Untreated WT and KO mice were used as negative controls, while human plasma was used as a positive control; **(D)** Tail-bleeding assay. The coagulation time was evaluated in neonatally-treated FIX KO mice (*n* = 5) and their *wild-type* littermates (*n* = 8). Data are shown as mean ± SEM and analyzed by one-way ANOVA with Tukey’s multiple comparison test.

**FIGURE 3:**
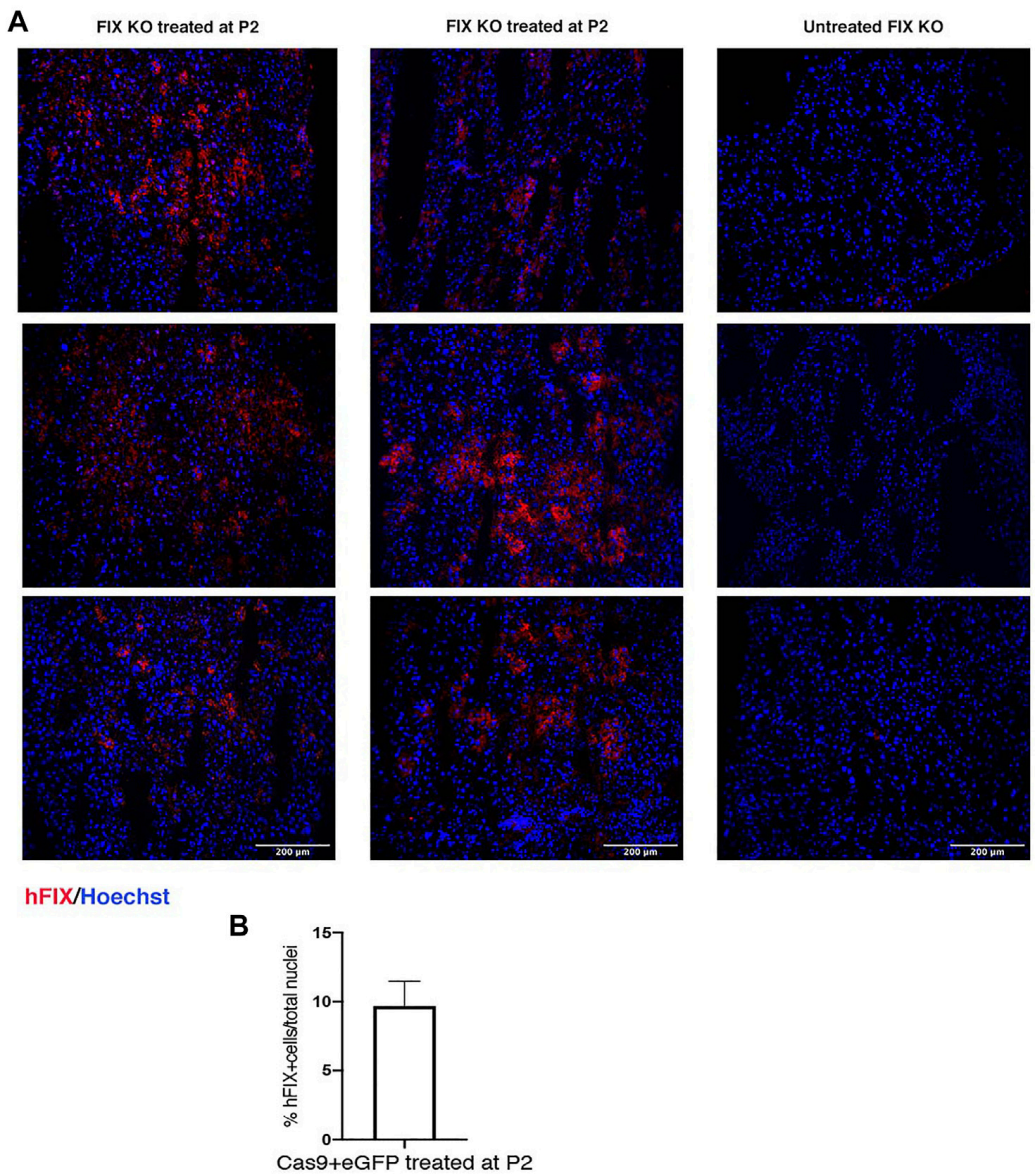
Immunohistochemical analysis of liver section. **(A)** hFIX protein was detected in liver sections of FIX KO mice treated at P2 with rAAV8-SaCas9 and rAAV8-donor-hFIX. Sections were stained with anti-hFIX antibody (red) and nuclei were counterstained with Hoechst (blue). Scale bar, 200 μm; **(B)** Quantification of hFIX positive cells normalized by total cell nuclei.

Next, to evaluate the therapeutic efficacy of the approach, we treated FIX KO male neonate mice (*n* = 12), a model of hemophilia B, and control littermates at P2 with rAAV8-donor-hFIX (2.0E11 vg/mouse) in combination with the SaCas9-sgRNA8 encoding vector [rAAV8-SaCas9-sgRNA8, 6.0E10 vg/mouse] (Figure 2A). This donor/SaCas9 vector ratio in wild-type mice resulted in the highest hFIX levels (HDR-L, Figure 1C). We first measured hFIX in plasma by ELISA and Western blot analysis. All treated mice showed hFIX plasma values that were about 100–150% of the human population values (Figure 2B). These results were similar to those obtained in the HDR-H group in WT mice (Figure 1C), which showed a reduction compared to the expected values present in the HDR-L-treated animals. Similar results were obtained by Western blot analysis, with FIX levels comparable to those present in the same volume of human plasma (Figure 2C). To assess the coagulation activity and, thus, the therapeutic efficacy of the treatment, we challenged treated FIX-KO mice by performing the tail clip test, which involves amputation of the tail tip and determination of the clotting activity by measuring the amount of blood loss over 20 min. We observed that the clotting capacity of FIX KO mice, when treated with rAAV8-donor-hFIX and AAV-SaCas9 vectors, was indistinguishable from that of WT animals (Figure 2D). Histological analysis of liver sections showed the presence of hFIX-positive cells in the treated mutant animals, while no signal was observed in the untreated control group (Figure 3A). Quantification of hFIX-positive cells showed a recombination rate of about 10% (Figure 3B). Interestingly, many positive cells were present in clusters suggesting that the recombination event occurred soon after viral transduction and the genetic modifications were inherited by the daughter cells, forming groups of hFIX-positive hepatocytes.

**FIGURE 4:**
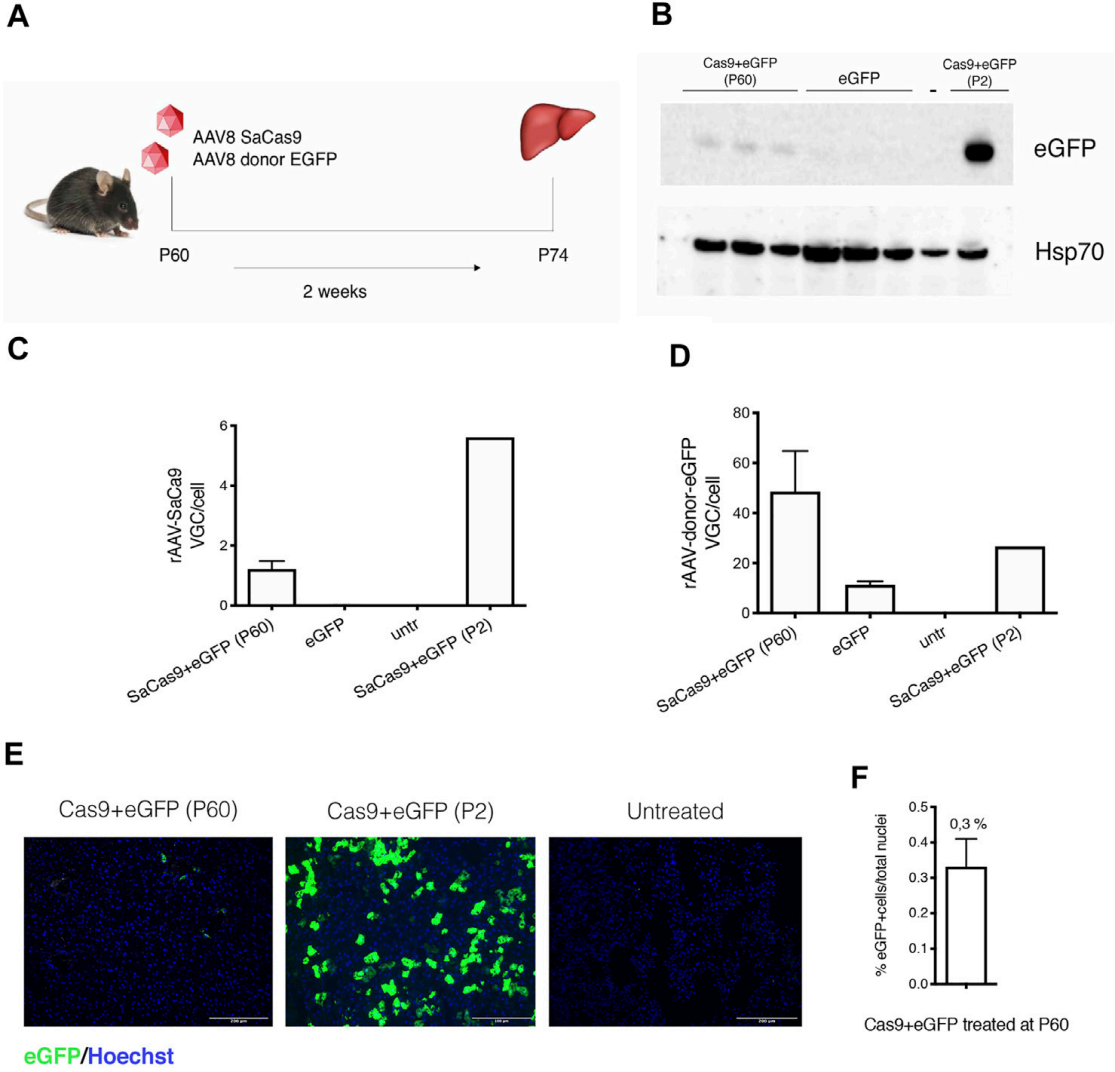
Gene targeting in adult wild-type mice liver with a reporter donor-eGFP. **(A)** Experimental scheme. WT mice were transduced with AAV8-SaCas9 (1.0E11 vg/mouse) and AAV8-donor-EGFP (5.0E11 vg/mouse) at postnatal day (P) 60 and their livers were collected 2 weeks post-injection (*n* = 3); **(B)** Western blot analysis of eGFP protein from liver extracts. In the analysis are included two samples from animals treated at P60 with donor-eGFP+Cas9, donor-eGFP (no Cas9), untreated *wild-type* mouse (−) and mouse treated with donor-eGFP+Cas9 at P2 (+); **(C,D)** rAAV8-SaCas9 and rAAV8-donor-eGFP viral genome copy analysis; **(E)** Immunohistochemical analysis of eGFP protein in mice treated with SaCas9 and donor-eGFP at P60 and P2. Nuclei were stained with Hoechst (blue). Scale bar, 200 μm; **(F)** Quantification of eGFP-positive cells normalized by the total number of cell nuclei.

We then tested the approach in adult animals. We first injected a GFP-reporter AAV (5.0E11 vg/mouse of AAV-eGFP-donor and 1.0E11 vg/mouse of AAV-SaCas9) in WT P60 mice and sacrificed them after 2 weeks (Figure 4A). Western blot analysis showed the presence of eGFP in the liver of treated animals, although at much lower levels than in animals injected at P2 (Figure 4B), while no signal was observed in untreated animals. Quantification of the viral genomes resulted in the expected levels, with higher VGC of the donor AAV-eGFP vector (Figures 4C,D). The percentage of eGFP-positive hepatocytes was about 0.33% (Figures 4E,F).

**FIGURE 5:**
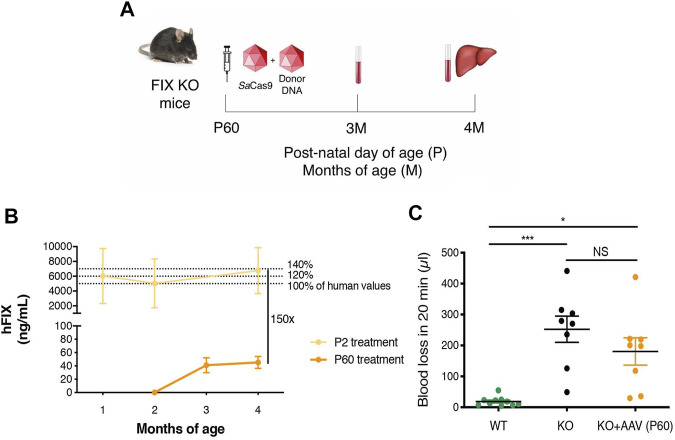
Gene targeting of adult FIX KO mice with gene-targeting strategy coupled to SaCas9. **(A)** Experimental plan. FIX KO adult mice were injected at 2 months of age (P60) with 1.0E11 vg/mouse of AAV8-SaCas9 and 5.0E11 vg/mouse of AAV8-donor-hFIX. Blood was collected 1 and 2 months after treatment. **(B)** Plasma hFIX levels in mouse transduced with *Sa*Cas9 and donor-hFIX at post-natal (P) day 2 (*n* = 15) and 60 (*n* = 8). hFIX concentration obtained from blood samples at months 1, 2, and 4 (P2 treatment) and 2, 3 and 4 months (P60 treatment). 5,000 ng/ml corresponds to the normal FIX plasma levels in healthy individuals in the human population. **(C)** Tail-bleeding assay. Bleeding time was evaluated in treated adult FIX KO mice (*n* = 8) and compared to untreated WT (*n* = 10) and FIX KO (*n* = 8) mice. Data are shown as mean ± SEM and statistically analyzed by one-way ANOVA with Tukey’s multiple comparison test.

To determine the efficacy of the approach in the adult diseased model, we injected FIX KO male animals of 2 months of age with 5.0E11 vg/mouse of AAV-FIX-donor and 1.0E11 vg/mouse of AAV-SaCas9 (Figure 5A). Mice were sacrificed 2 months after AAV dosing. Determination of hFIX in plasma by ELISA indicated that the treatment resulted in very low plasma FIX levels, in the range of 50 ng/ml (about 1% of normal values, Figure 5B), which were about 150-fold lower than those detected in animals injected at P2. The tail-clip test showed a non-statistically significant reduction in blood loss, compared to untreated FIX KO mice (Figure 5C), while blood loss in control WT mice was minimal.

**FIGURE 6:**
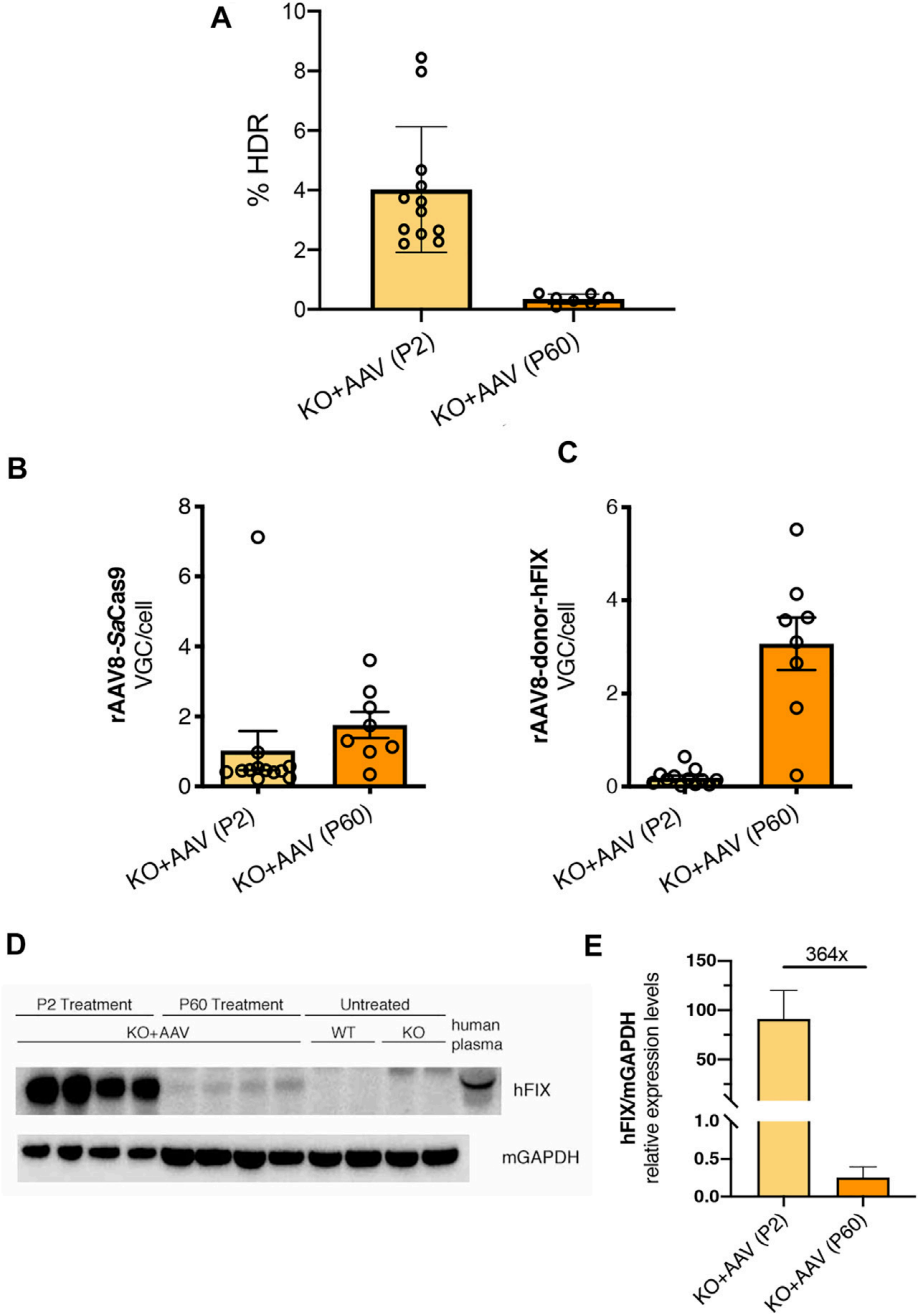
Evaluating the genome targeting rate, the presence of viral genome copies and hFIX protein in hepatocytes. **(A)** Genome targeting rate was evaluated in neonatal and adult-treated mice by ddPCR of liver genomic DNA (from Figures 2, 5); **(B,C)** rAAV8-SaCas9 and rAAV8-donor-hFIX viral genome copies analysis from FIX KO mice treated at P2 and P60 (from Figures 2, 5); **(D)** Western blot analysis of hFIX protein in liver extracts. Mice treated at P2 and P60, together with untreated WT and KO were analyzed. Human plasma was used as a positive control; **(E)** Western blot was quantified and normalized with housekeeping GAPDH protein.

To understand the reasons for the lower efficiency of the approach in adult FIX animals, we first evaluated the rate of targeting by ddPCR in both P2- and P60-treated groups. The gene-targeting rate for neonatal and adult injected groups was 4.02 and 0.35%, respectively, (Figure 6A). Next, we assessed transduction efficiency by determining the VGC per cell in treated livers and compared these results with those obtained for P2 injected animals. We observed that both AAV vectors were present in the hepatocytes, at levels in line with the applied dose (Figures 6B,C). In spite that the AAV dose used at P2 was higher than that one used at P60, the VGC values present in the animals injected at P2 were lower than those of the P60 group, probably due to vector DNA loss, not occurring in the adult-injected group. We observed that the loss of AAV-FIX-donor DNA in P2 treated animals was more pronounced compared to the relative loss of the AAV-SaCas9 vector in the same animals.

Next, we performed a Western blot analysis of liver protein extract (Figure 6D). We observed a clear signal corresponding to hFIX protein in adult treated animals, which was of lower intensity than the one present in P2-injected mice. However, quantification of the blots indicated that the difference in hFIX levels between mice injected at P2 and P60 was about 364-fold (Figure 6E), suggesting lower hFIX levels in the P60 group than those observed in plasma by ELISA (Figure 5B, about 150-fold).

**FIGURE 7:**
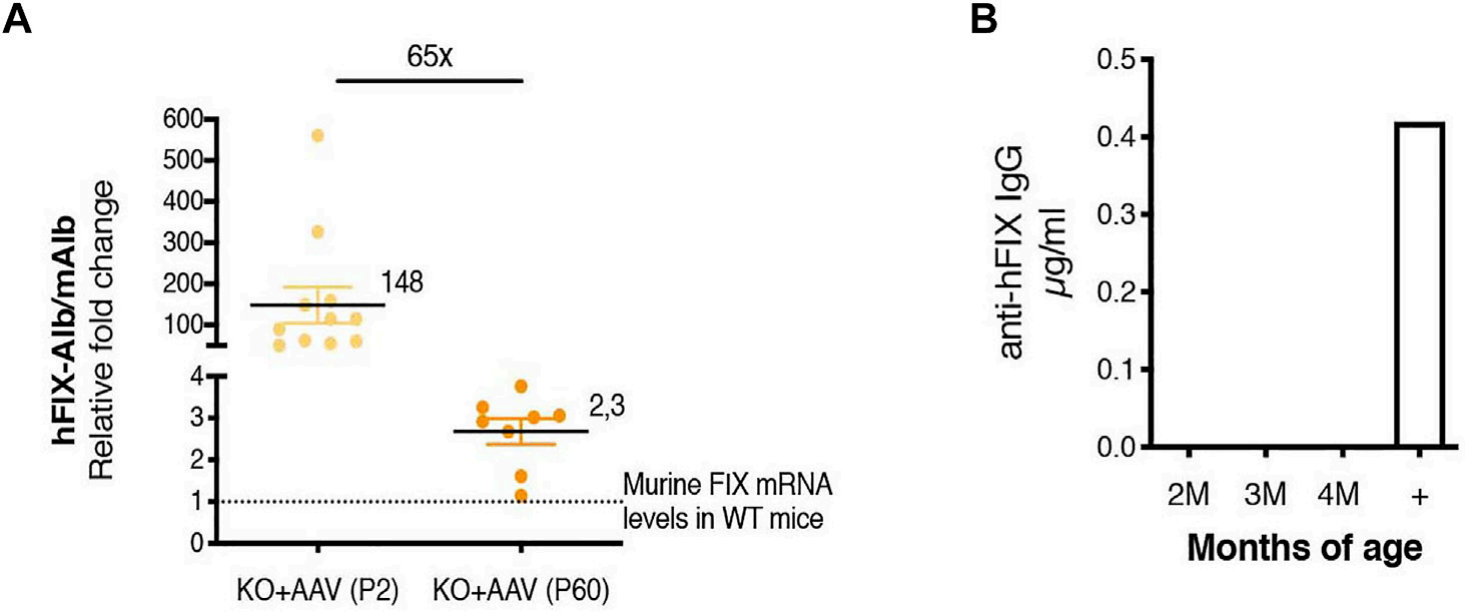
Chimeric Alb-hFIX mRNA expression levels in mice treated at P2 and P60 and evaluation of anti-hFIX neutralizing antibodies. **(A)** Analysis of hybrid Alb-hFIX mRNA expression levels. Alb-hFIX mRNA expression was evaluated in both treated groups (P2, *n* = 10, and P60, *n* = 8) and endogenous mFIX mRNA was analyzed in wild-type mice. The fold-increase between hFIX in treated mice and endogenous mFIX in WT mice is indicated; **(B)** Anti-hFIX neutralizing antibodies were measured using an ELISA assay. Mice plasma was analyzed at different time-points by incubation with hFIX recombinant protein. An anti-hFIX antibody was used as a positive control.

We also compared the levels of the chimeric mAlb-hFIX mRNA to those of the endogenous mFIX mRNA (Figure 7A). We calculated the efficiency of the different sets of primers used for the comparison by serial dilution of the template DNA. We observed that their efficiency was similar, in the range of 100% (Supplementary Figure S1). The comparison of the chimeric mRNA in P2-injected mice vs the P60 group confirmed the results observed in the ELISA and WB experiments (Figures 5B, 6D,E), with a difference of about 65-fold (Figure 7A). Importantly, we observed that the levels of the chimeric mRNA, both in mice injected at P2 and P60, were much higher than the levels of mFIX present in a WT adult animal ∼148- and ∼2.3-fold, respectively). This result may indicate that only a fraction of the chimeric mAlb-hFIX mRNA was indeed efficiently translated into hFIX protein. In order to assess whether the low values of hFIX were related to the presence of anti-hFIX IgG antibodies, we performed an ELISA assay. We did not detect anti-hFIX IgG antibodies (Figure 7B).

## Discussion

Gene therapy by gene replacement approaches for hemophilia B showed promising results in clinical trials (Nathwani et al., 2011; George et al., 2017; George et al., 2020). However, its application in neonatal or pediatric settings, characterized by a growing liver, is limited by vector DNA loss associated with hepatocyte duplication (Bortolussi et al., 2014; Cunningham et al., 2008; Wang et al., 2012). Hemophilia represents an ideal target for genome-editing approaches, as minor increases in the levels of circulating factor IX can have an important impact in correcting the symptoms (White et al., 2001). In our previous work, we showed that the GeneRide strategy (Barzel et al., 2015) coupled to the CRISPR/Cas9 platform efficiently targets the therapeutic transgene to the albumin locus in a Crigler-Najjar mouse model, leading to the complete rescue from neonatal lethality and phenotype abnormalities (De Caneva et al., 2019). Importantly, this strategy is efficiently applicable to neonatal mice where hepatocytes are actively proliferating and the homologous directed repair (HDR) mechanism is active (Xue and Greene, 2021).

Different genome editing/targeting strategies have been applied to hemophilia mouse models, ranging from the insertion of an hFIX cDNA into the murine FIX locus (Wang et al., 2019) to the correction of mutations previously generated in the endogenous FIX locus (Guan et al., 2016; Huai et al., 2017; Ohmori et al., 2017). Here, aiming at developing an efficient strategy for hemophilia B, we first treated neonate P2 wild-type and FIX KO mice with a dual rAAV vector strategy: one expressing the SaCa9/sgRNA, and one containing the donor-hFIX cDNA flanked by arms of homology for the albumin locus. This strategy has a series of advantages, such as the permanent insertion of the therapeutic cDNA downstream of the albumin ORF, without affecting albumin gene expression (De Caneva et al., 2019). Moreover it is potentially applicable to the entire mutational spectrum of the hFIX gene, not possible with mutation-specific approaches (Guan et al., 2016; Huai et al., 2017; Ohmori et al., 2017). Previously reported gene targeting strategies for hemophilia B inactivate the albumin allele (Sharma et al., 2015; Wang et al., 2020), raising concerns related to the potential impact on albumin production. We have seen that the insertion of the hFIX cDNA is stably transmitted to daughter cells upon duplication, as supported by the presence of hFIX-positive hepatocyte clusters in liver sections (Figure 3), and by the transgene constant levels even after partial hepatectomy (Barzel et al., 2015). The dual AAV proposed approach appears to be safe, as no signs of tumorigenesis induction, liver inflammation, or changes in plasma albumin levels were observed (De Caneva et al., 2019). The specificity and the absence of off-target activity of the approach used here and in previous studies were analyzed by amplifying and sequencing on- and off-target predicted amplicons (De Caneva et al., 2019). Importantly, in the case that the DSB is repaired by the error-prone NHEJ DNA repair mechanism instead of the desired and precise HDR mechanism, potential risks of damaging the 5′ splice site and, consequently, affecting albumin gene expression are limited since the sgRNA target site is located in the downstream intron (De Caneva et al., 2019). Complete inactivation of the hypomorphic targeted allele was observed when a gene editing strategy for ornithine transcarbamylase deficiency was tested in adult Spf-Ash mice, as the DSB was corrected mainly by NHEJ, worsening the phenotype of treated mice (Yang et al., 2016). We expect that most of the vectors expressing the SaCas9 nuclease will get lost during hepatocyte proliferation (Cunningham et al., 2008; Wang et al., 2012; Bortolussi et al., 2014) limiting safety concerns associated with long-term expression of the nuclease. Other strategies to obtain transient expression of the SaCas9 vector, such as mRNA or protein delivery, or self-limiting circuits (Ramakrishna et al., 2014; Yin et al., 2016; Petris et al., 2017; Finn et al., 2018), could further increase the overall safety of the procedure.

In neonate-dosed animals, we have shown both efficacy and long-term stability of the treatment. ELISA quantification showed supraphysiological levels of circulating hFIX, and, in some animals, more than 200% of normal human values were observed. Notwithstanding the high levels of hFIX carrying the V86A, E277A, and R338L-Padua hyperactive mutations (Simioni et al., 2009; Lin et al., 2010; Kao et al., 2013), we observed no evident adverse effects or mortality in the treated animals. It was reported that this triple-mutant hFIX version presents a 15-fold increase in activity compared to the wild-type form (Kao et al., 2013). In line with the expected enhancement of gene targeting in the presence of a double-strand DNA break (Rouet et al., 1994), hFIX levels in the presence of nuclease in the neonatal-treated animals were increased up to 100-fold, compared to the group without nuclease. To test the functional rescue of the phenotype we performed the tail-clip test. Neonatal-treated mutant mice showed similar coagulation capacity compared to WT animals.

Our previous work, using a reporter eGFP cDNA in neonatal mice, shows that using a SaCas9/sgRNA that targets the intron downstream to the exon containing the stop codon of the albumin gene, we can achieve up to 15% of recombinant hepatocytes, with animals reaching up to 24% of eGFP-positive cells (De Caneva et al., 2019). Here, neonatal treatment of hemophilia B mice with a donor construct containing the hFIX cDNA resulted in 4–10% of recombinant hepatocytes, as determined by ddPCR and quantification of liver sections. However, after treating adult mice, both the eGFP and hFIX levels observed were considerably lower than those of the neonate treatment. In fact, we detected ∼0.3% of eGFP-positive hepatocytes in adult-treated mice. A similar value was observed in adult FIX KO mice treated with the AAV-FIX-donor DNA (0.35%, determined by ddPCR). The treatment of adult FIX KO was not efficient in increasing hFIX to therapeutic levels. In fact, treated mice had an increase of about 1% of plasma hFIX while coagulation activity after the tail clip test was not statistically different from the one observed in untreated FIX KO mice. The lower efficacy observed in adult animals in the presence of nucleases could be related to the post-mitotic condition of the adult liver, in which DNA damage is mainly corrected by NHEJ and not by HDR (Xue and Greene, 2021), limiting the overall gene targeting efficiency, as already observed in other models (Yang et al., 2016). On the contrary, in the absence of nucleases we observed similar gene targeting rates in neonate and adult treatments (Barzel et al., 2015). While HDR occurs in proliferating cells in the S/G2 phase of the cell cycle, the majority of AAV-HR events occurred in non-proliferating hepatocytes in juvenile mice (Tsuji et al., 2022). Thus, it is very probable that important differences in the mechanisms exist when comparing genome targeting in the presence or in the absence of nucleases. A direct comparison between P2 and P60 treatments, using the same AAV doses, both in the presence and absence of nucleases, should shed light to this issue. Another concurrent reason could be related to the lower AAV dose used in the adult treatment. While the AAV doses successfully used in neonatal mice were similar to those used by Wang et al. (Wang et al., 2020), in the case of adult animals we have used 5.0E11 vg of AAV-FIX-donor and 1.0E11 vg of AAV-SaCas9 (2.5E13 vg/kg and 5.0E12 vg/kg, respectively), similar to the one used by Yang et al. in SpfAsh mice (Yang et al., 2016), a dose that was double of the one we used for the neonate treatment (2.5E14 vg/kg and 3.0E13 vg/kg, for donor and SaCas9 vectors, respectively), but it was about 50-fold lower than that successfully used in adult FIX animals by Wang et al. (Wang et al., 2020). This comparison suggests that further optimization of the vector dose may result in a successful rescue also in adult-treated mice. However, differences in recombination rate may be also affected by the targeting position in the albumin gene (1st intron in Wang et al., vs. exon 14 in our approach). Others have also used a similar approach in adult animals targeting the ApoA1 gene with AAV doses that were approximately 50× higher than the one in our study (De Giorgi et al., 2021).

We evaluated the mRNA expression levels of the hFIX-Alb chimeric mRNA both in the neonate and adult-treated animals and compared them to the endogenous FIX expression levels present in wild-type animals. Unexpectedly, the hFIX mRNA levels in both groups of treated animals were much higher than the protein levels detected by ELISA (Figures 5B, 7A). To shed light on the reasons for this difference, we analyzed the hFIX levels in liver protein extracts, which did not present an abnormal accumulation of hFIX in hepatocytes. Further, we failed to detect anti-hFIX antibodies in the plasma of treated animals at any of the analyzed time points (Figure 7B). Thus, we hypothesize that one of the possible reasons for the differences between observed mRNA and protein levels could be a less efficient protein translation of the hFIX ORF in the chimeric albumin-hFIX mRNA. The presence of 2A peptides between two ORFs results in non-stoichiometric levels of the second ORF, although the teschovirus-1 2A peptide presents the highest efficiency among the different 2A peptide variants, reaching up to 85% in the liver using a construct coding for EGFP-P2A-mCherry (Kim et al., 2011). However, the skipping efficiency may be different with other ORFs (Chng et al., 2015; Liu et al., 2017). Another possibility could be the read-through without ribosomal skipping. We believe that this possibility should be ruled out since in the P60-treated animals we were not able to detect a higher molecular weight band corresponding to the fused albumin-hFIX protein in the Western blot analysis of liver extracts. A band of 130 kDa detected in the P2-treated animals was too faint to account for an inefficient ribosomal skipping (Supplementary Figure S2). We have also previously shown the absence of inflammation in the liver in a Crigler-Najjar mouse model that was treated with the dual AAV strategy (De Caneva et al., 2019). Further experiments will be required to fully understand the nature of these unexpected results.

To summarize, here we presented results supporting a gene targeting approach targeting the albumin gene exploiting the use of the CRISPR/SaCas9 platform. This strategy can potentially be applied for the treatment of pediatric patients suffering from hemophilia and other liver monogenic diseases of the liver, by simply replacing the therapeutic gene. For the treatment of adult patients, further studies for the improvement of targeting efficiency are still required.

## Data Availability

The original contributions presented in the study are included in the article/Supplementary Material, further inquiries can be directed to the corresponding author.
