# Supplementary figures and images for: Promoterless Gene Targeting Approach Combined to CRISPR/Cas9 Efficiently Corrects Hemophilia B Phenotype in Neonatal Mice
Source: Front Genome Ed. 2022 Mar 11;4:785698. doi: 10.3389/fgeed.2022.785698 (PMC8962648; doi:10.3389/fgeed.2022.785698)

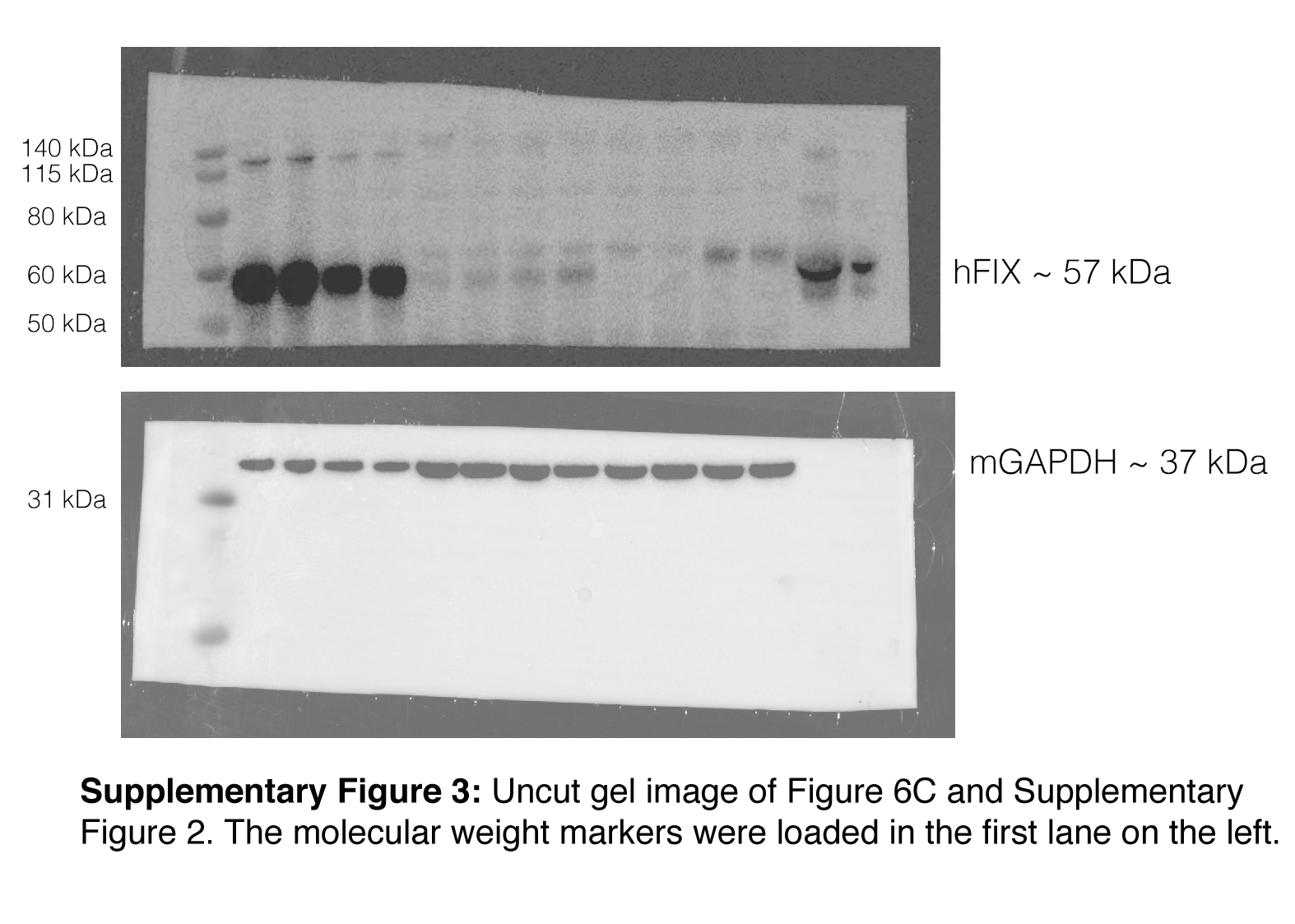

Supplement: Supplementary file 1 [file Image3.tiff]

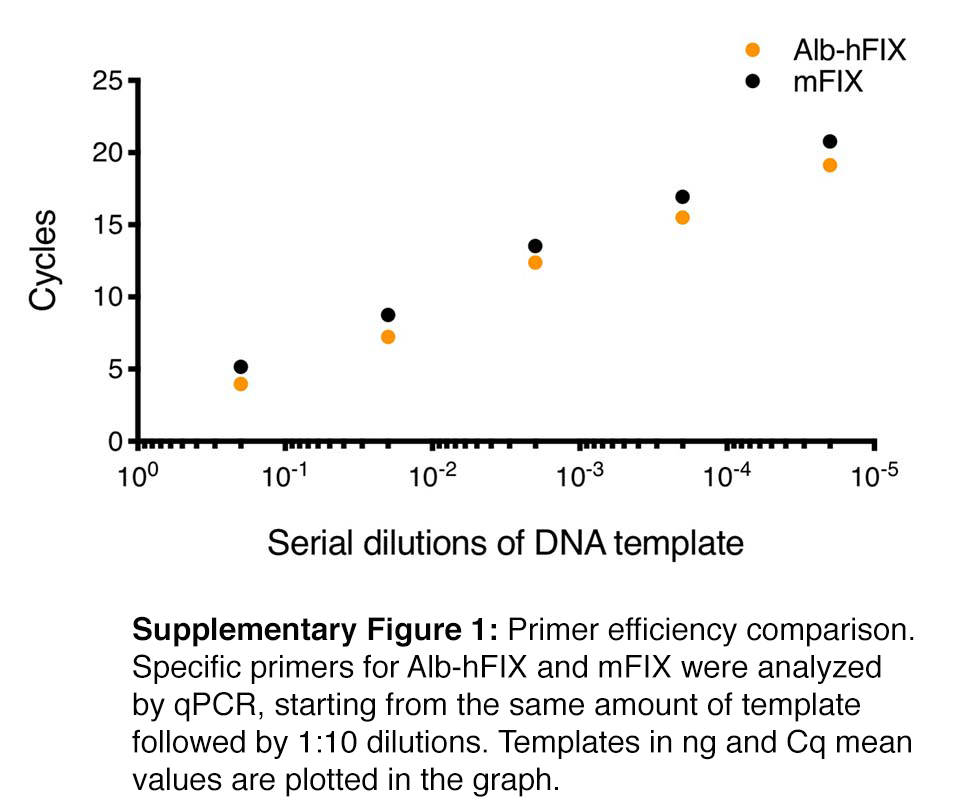

Supplement: Supplementary file 2 [file Image1.tiff]

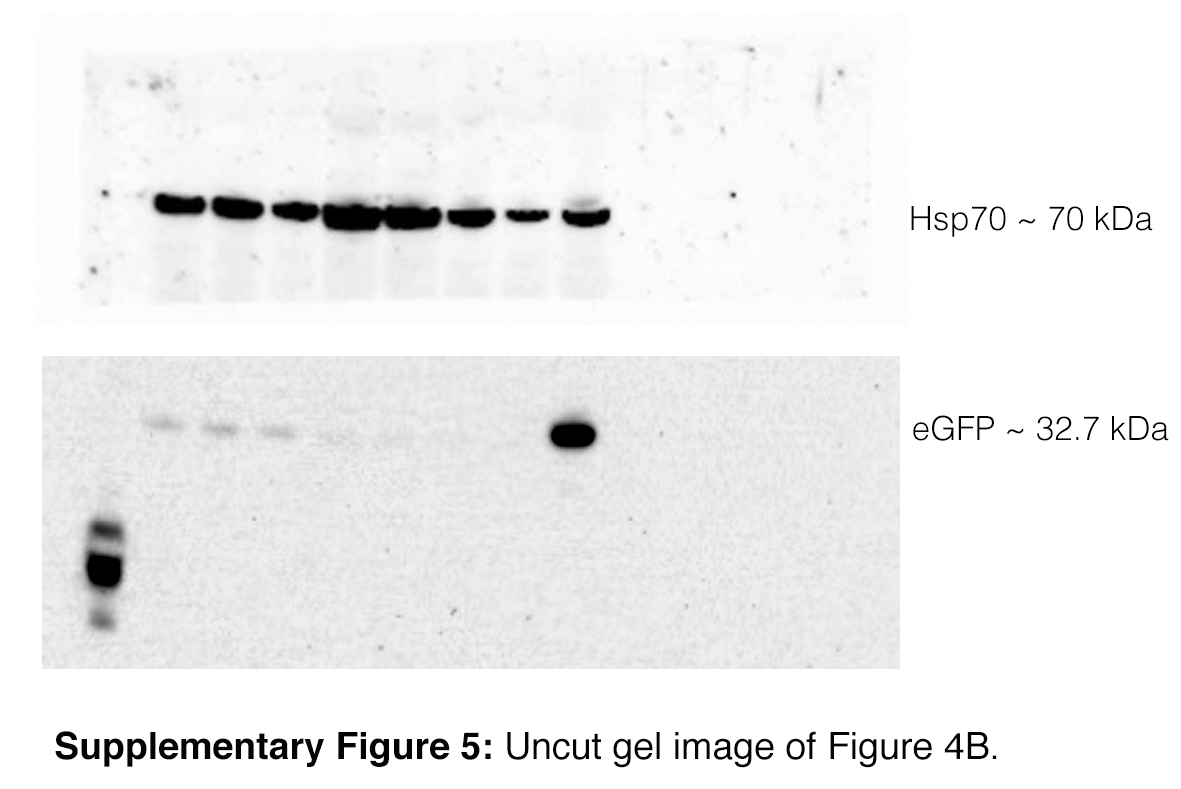

Supplement: Supplementary file 4 [file Image5.tiff]

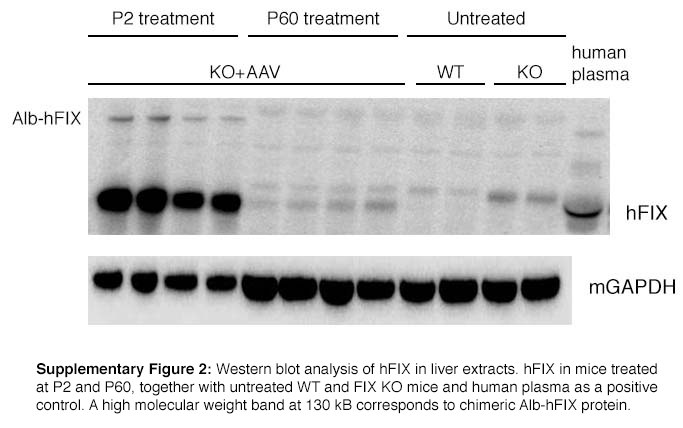

Supplement: Supplementary file 5 [file Image2.tiff]

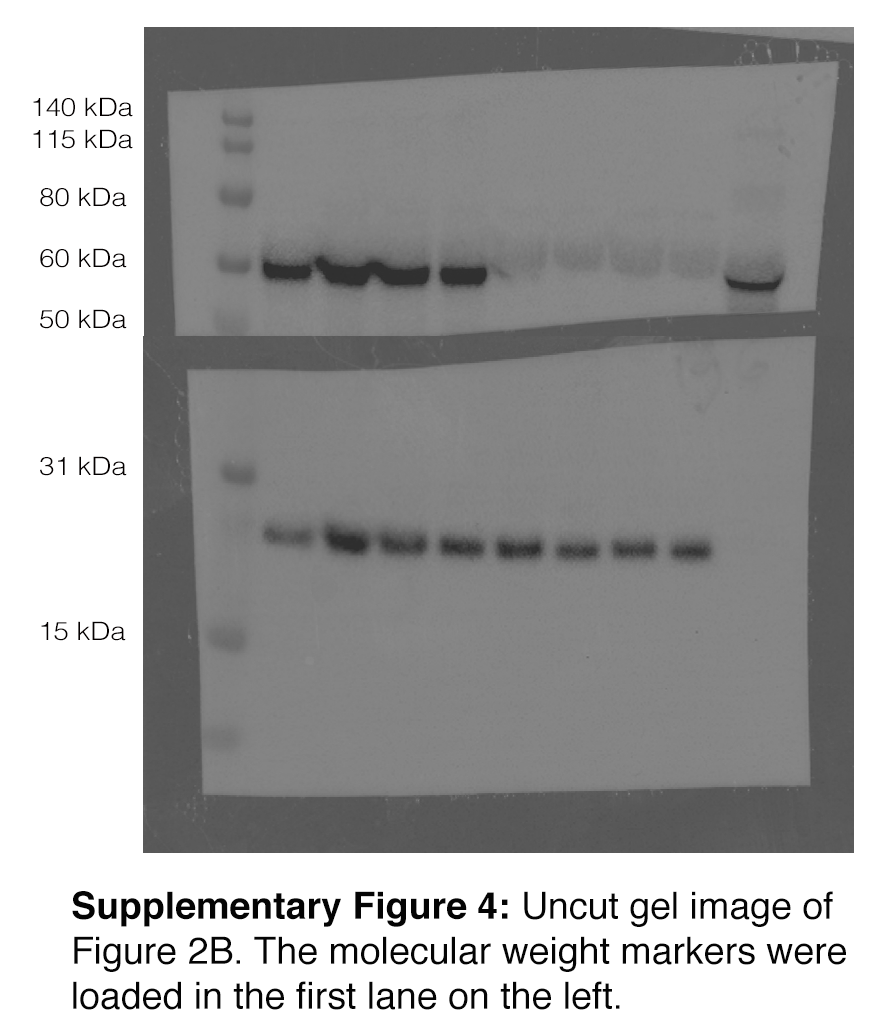

Supplement: Supplementary file 6 [file Image4.tiff]
