# Supplementary material for: Promoterless Gene Targeting Approach Combined to CRISPR/Cas9 Efficiently Corrects Hemophilia B Phenotype in Neonatal Mice
Source: Front Genome Ed. 2022 Mar 11;4:785698. doi: 10.3389/fgeed.2022.785698 (PMC8962648; doi:10.3389/fgeed.2022.785698)
Supplement: Supplementary file 3 [file Table1.pdf]

**Target protein**	**Supplier**	**Source**	**Dilution**
eGFP	Santa Cruz	Rabbit	1/3000
GAPDH-HRP	Sigma-Aldrich	Mouse	1/10000
hFIX	Affinity Biologicals	Goat	1/3000

Supplementary Table 1: Primary and secondary antibodies used.
